# Risk Factors for Spinal Cord Monitoring Alerts in Paediatric Patients Undergoing Spinal Deformity Surgery

**DOI:** 10.7759/cureus.115608

**Published:** 2026-09-01

**Authors:** Michelle Lee, Stuart James, Dimitrios Kefalas, Alwyn Jones, Sashin Ahuja, Nazeer Moideen, John Howes, Michael J McCarthy

**Affiliations:** 1 Department of Trauma and Orthopaedics, Cardiff Medical School, Cardiff, GBR; 2 Welsh Centre for Spinal Surgery and Trauma, Cardiff and Vale University Health Board, Cardiff, GBR; 3 Department of Neurosurgery, Cardiff and Vale University Health Board, Cardiff, GBR

**Keywords:** intraoperative neuromonitoring, paediatric scoliosis, risk factors, spinal deformity surgery, surgical interventions

## Abstract

Background: Intraoperative neurophysiological monitoring (IONM) is a standard practice during spinal deformity surgery, but evidence in paediatric patients is limited. This retrospective study aims to identify factors associated with IONM alerts during paediatric spinal deformity surgery.

Methods: Patients ≤18 years old who underwent scoliosis correction surgery in a single institute between 2017 and 2022 were reviewed. Analysed factors included estimated blood loss (EBL), operative time, pre- and postoperative major Cobb angles, and mean corrective rate.

Results: Among 216 cases, eight had significant IONM alerts. These patients had a significantly longer operative time (p = 0.030), preoperative major Cobb angle (p = 0.048) and postoperative major Cobb angle (p = 0.019). Age (p = 0.481), sex (p = 0.416), mean corrective rate (p = 0.799) and EBL (p = 0.062) did not differ significantly. Alerts were found to be primarily triggered by rod application and curve correction (62.5%), screw insertion (25.0%) and corpectomy (12.5%). In the alert group, no patients experienced postoperative neurological deficits, and one operation (12.5%) was aborted. One patient (0.5%) in the non-alert group developed a transient postoperative foot drop but recovered completely nine weeks later.

Conclusion: Greater operative time and preoperative and postoperative major Cobb angles were associated with IONM alerts. Most alerts occur during and after the insertion of instrumentation and curve correction manoeuvres. The triggering event should be reversed immediately, and if signals do not recover, surgery should be aborted and staged to prevent neurological deficit.

## Introduction

With every spinal deformity surgery, there is a 0.5-3.2% risk of major neurological complications [1]. In 1992, the Scoliosis Research Society advocated the use of intraoperative neurophysiological monitoring (IONM) or spinal cord monitoring during spinal surgery to detect iatrogenic neurological injury at an earlier reversible stage [2]. Over the past decade, several studies have demonstrated the efficacy and vitality of IONM, and it has now become the standard of care during spinal deformity surgery [3-6]. The British Society of Clinical Neurophysiology recommended the use of IONM in all spinal deformity surgeries; in particular, the need for motor evoked potentials (MEPs) and somatosensory evoked potentials (SSEPs) in high-risk cases [7]. The most recent guidelines published in October 2018 have upgraded the recording of transcranial muscle MEPs from guideline to standard, and included peripheral nerve recording as a technical control during SSEP recording [8].

There are several modalities of IONM that can measure the functional integrity of the spinal cord during surgical deformity corrective procedures. The first and most widely employed approach consists of recording SSEPs in response to electrical stimulation of the lower limb peripheral nerves, typically the posterior tibial nerves. SSEPs were first introduced in the early 1970s and could reliably monitor the integrity of the somatosensory pathways in the dorsal columns. However, the use of SSEPs has its limitations as it does not provide any information regarding the corticospinal tract. This led to the introduction of muscle MEPs under general anaesthesia in 1993, which can detect injuries to portions of the spinal cord supplied by the anterior spinal artery [9]. MEPs are very sensitive indicators of corticospinal tract injuries by recording muscle responses following a brief multi-pulse transcranial electrical stimulation of the primary motor cortex. The use of both SSEPs and MEPs has demonstrated exceedingly high sensitivity and specificity in detecting early spinal cord dysfunction [10,11].

Although IONM is widely used in spinal surgery, there is limited literature related to its use in paediatrics when compared to adults. This study aims to identify clinical factors that are associated with IONM changes in paediatric patients.

## Materials and methods

Subjects

A database of all patients who underwent spinal surgery at our institution was filtered to include those who had scoliosis correction surgery between 2017 and 2022 and were ≤18 years old at the time of surgery. Patient medical records with inadequate information regarding important variables were excluded. All patients included in the study were categorised into either the alert or non-alert group. The alert group comprised all patients who received a neuromonitoring alert during the operation. In contrast, the non-alert group consisted of all patients who did not experience a neuromonitoring alert.

Intraoperative neurophysiological methodology

IONM was used in all cases, including SSEPs, MEPs and electromyography (EMG). SSEP and MEP baselines were recorded after induction of anaesthesia and final positioning, and before the beginning of the procedure. SSEPs and MEPs were monitored when possible and reasonable, with monitoring continued for at least 20 minutes after completing instrumented fixation of the spine. The alert criteria used for SSEPs included a 50% reduction in amplitude or a 10% increase in latency compared to their baselines. For MEPs, the alert criterion used was an 80% reduction in amplitude compared to their baselines.

Lower limb SSEPs were elicited using bilateral stimulation of the posterior tibial nerve. Recording was performed with the use of corkscrew-like needle electrodes positioned on the scalp, and subdermal straight needles or surface electrodes on the popliteal fossa. Upper limb SSEPs were elicited with stimulation of the median or ulnar nerve and recorded using corkscrew electrodes placed on the scalp. The transcranial stimulations for MEPs were delivered with the use of corkscrew-like needle electrodes and recorded using subdermal needle electrodes positioned over two or more muscles of the lower limbs (usually tibialis anterior and abductor hallucis) and one muscle of each hand (usually abductor pollicis brevis or abductor digiti minimi). Total intravenous anaesthesia (TIVA) technique was used, which consisted of continuous infusion of propofol and remifentanil with short-lasting muscle relaxants, for intubation purposes only. Halogenated agents and nitrous oxide were avoided during the operations.

Response to IONM alert

In the event of an IONM alert, the surgeons and the anaesthesiologist were notified and initiated the standard protocol for investigation and intervention of IONM alerts. This included a series of checks, which included the level of spine operated on, blood pressure, level of anaesthesia, temperature and other factors that could affect IONM signals. After ruling out technical issues (i.e., IONM equipment and/or electrode faults), positional issues (i.e., peripheral nerve compression) and anaesthetic issues (i.e., changes in depth of anaesthesia), the alert was deemed to be a result of potential spinal cord injury. This warranted further interventions after prompt decision-making by the spinal surgeons. Surgical procedures would typically be reversed, including the removal of metalwork or reversal of curve correction manoeuvre and verifying that there was no breach or compression of the spinal cord caused by bone, haematoma or instrumentation. If signals could not be restored to baseline despite interventions performed, a decision would be made by the surgical team to abort and stage the operation to a later date. Patients were examined for neurological deficits, and imaging modalities, including CT or MRI of the spine, were used to aid staged surgery.

All patients were monitored for neurological deficits immediately upon waking up and six weeks postoperatively. The nature of the alerts was defined and categorised (Table 1).

**Table 1:** Definitions of types of alerts

Type of alert	Definition
True positive	(1) An alert with recovery of potentials following corrective interventions (i.e., release of instrumentation, release of curve correction, increased blood pressure), without postoperative neurological deficit. These were considered reversible or transient true positive alerts. (2) An alert without recovery of potentials despite performing interventions, and with the patient having new postoperative neurological deficits
False positive	An alert without recovery of potentials after performing interventions followed by no new postoperative neurological deficits
True negative	(1) No alert and no development of postoperative neurological deficits. (2) An alert with recovery of potentials caused by factors that did not involve potential damage to the spinal cord. This included positional issues (i.e., peripheral nerve compression, vascular insufficiency due to occlusion), technical issues (i.e., detachment of electrodes, electrical interference or artefact) or anaesthetic issues (i.e., use of inhalation agents)
False negative	No alert but patient developed new postoperative neurological deficits

Variables

Demographic, clinical and intraoperative data, including age, sex, type of scoliosis, estimated blood loss (EBL) and operative time, of all patients were recorded. Radiographic measurements of preoperative major Cobb angle, postoperative major Cobb angle and degree of correction were measured and calculated at the time of the study. Preoperative and postoperative major Cobb angles were measured on standing full-spine radiographs using the standard Cobb method. The major curve was defined as the largest structural curve. In the alert group, mean arterial pressure (MAP) at the time of alert, timing of the alert, trigger events that caused IONM alerts and the interventions performed were recorded.

Statistical analysis

The distribution of continuous variables was assessed using the Shapiro-Wilk test and visual inspection of Q-Q plots. Equality of variances was assessed using Levene's test. Normally distributed continuous variables with equal variances (preoperative major Cobb angle) were compared using an independent-samples Student's t-test. Non-normally distributed variables (age, postoperative major Cobb angle, mean corrective rate, operative time and EBL) were compared using the Mann-Whitney U test. Fisher's exact test was used to analyse categorical variables (sex). Given the small number of true positive IONM alerts, results were interpreted cautiously. The potential for type I error arising from multiple comparisons was also considered, particularly for borderline statistically significant findings. Statistical analyses were performed using IBM^®^ SPSS^®^ Statistics version 27 (IBM Corp., Armonk, NY, USA). Statistical significance was set at p <0.05, with all tests being two-sided.

## Results

Patient characteristics

A total of 232 cases were found in the database, of which 16 were excluded because of inadequate medical records, leaving 216 cases (93.1%). All surgical episodes were found to be unique, with no repeat patients identified. The mean age at the time of surgery was 14.1 years. Patient characteristics are summarised in Table 2. The majority of the patients were diagnosed with adolescent idiopathic scoliosis (AIS) (71.8%), followed by neuromuscular scoliosis (13.4%), syndromic scoliosis (9.7%), congenital scoliosis (3.2%) and Scheuermann’s kyphoscoliosis (1.9%). The preoperative mean Cobb angle of the major curve was 64.0 ± 19.2°.

**Table 2:** Patient characteristics Diagnosis is represented as n (%). Preoperative major Cobb angle is presented as mean ± standard deviation (SD).

Patient characteristic	Value
Total no. of patients	216
Mean age	14.1
Sex	
Female	162
Male	54
Diagnosis	
Adolescent idiopathic scoliosis	155 (71.8%)
Neuromuscular scoliosis	29 (13.4%)
Syndromic scoliosis	21 (9.7%)
Congenital scoliosis	7 (3.2%)
Scheuermann’s kyphoscoliosis	4 (1.9%)
Preoperative major Cobb angle (°)	64.0 ± 19.2

Comparison between alert and non-alert groups

Out of the 216 patients, eight patients (3.7%) underwent eight operations for spinal deformity and experienced a true positive IONM alert intraoperatively. Among the perioperative factors, operative time (p = 0.030) was significantly greater in the alert group compared to the non-alert group. The preoperative major Cobb angle (p = 0.048) and postoperative major Cobb angle (p = 0.019) were also found to be larger in the alert group. However, age, sex, mean corrective rate and EBL did not demonstrate significant differences between the two groups (Table 3). Missing data were present for multiple variables, particularly operative time, as these variables were not routinely recorded in the earlier cases. Patients with missing data for a specific variable were excluded from the analysis of that variable. Type of scoliosis and incidence rates of IONM alerts were identified (Table 4). Incidence of IONM alerts was highest in patients diagnosed with congenital scoliosis. 

**Table 3:** Demographic factors, perioperative factors and postoperative results by group EBL, estimated blood loss; OR, odds ratio. Age, postoperative major Cobb angle, mean corrective rate, operative time and EBL were analysed using Mann-Whitney U test and presented as median (25th-75th percentile). Sex was analysed using Fisher’s exact test and presented as n (%). Preoperative major Cobb angle was analysed using Student's t-test and presented as mean ± standard deviation (SD). *p < 0.05, indicating statistical significance.

Variable	No alert	N	Alert	N	Statistical test	Statistical value	p-Value
Age (years)	14.0 (13.0-15.0)	208	13.5 (13.0-15.8)	8	Mann-Whitney U	U = 711.5	0.481
Sex (F:M)	157 (75.5%):51 (24.5%)	208	5 (62.5%):3 (37.5%)	8	Fisher's exact	OR = 1.8	0.416
Preoperative major Cobb angle (°)	63.3 ± 18.8	193	76.9 ± 22.5	8	Student's t-test	t = -1.9	0.048*
Postoperative major Cobb angle (°)	30.0 (23.75-40.25)	182	51.5 (33.0-55.0)	8	Mann-Whitney U	U = 372	0.019*
Mean corrective rate (%)	30.0 (17.75-39.25)	178	33.0 (15.0-42.0)	8	Mann-Whitney U	U = 674	0.799
Operative time (hours)	2.75 (2.5-3.0)	121	3.0 (3.0-3.0)	8	Mann-Whitney U	U = 275	0.030*
EBL (mL)	750 (350-750)	173	750 (750-1250)	8	Mann-Whitney U	U = 439	0.062

**Table 4:** Diagnoses by group

Diagnosis	Non-alert	Alert	Incidence of alerts (%)
Adolescent idiopathic scoliosis	151	4	2.6
Neuromuscular scoliosis	28	1	3.6
Syndromic scoliosis	20	1	5
Congenital scoliosis	5	2	40
Scheuermann’s kyphoscoliosis	4	0	0
Total	208	8	

Four operations (1.9%) had IONM alerts that were deemed true negatives, as they were caused by positional issues. Monitoring signals returned to baseline following repositioning in all cases. Three patients who were diagnosed with cerebral palsy had poorly formed or absent neuromonitoring signals preoperatively. They underwent operations with no IONM alerts, except for one of the three patients who experienced a true positive alert during rod application and curve correction.

Causes of IONM alerts

Causes of IONM alerts during all eight operations were identified. Five cases (62.5%) were caused by rod application and curve correction manoeuvre, two (25.0%) were caused by insertion of screws and one (12.5%) was caused by the corpectomy procedure (Table 5). Interventions included removal of instrumentation in three patients (37.5%), release of curve correction in two patients (25.0%), raising blood pressure in two patients (25.0%) and one operation was aborted and staged (12.5%) (Table 6). Aside from the abandoned case, all signals were recovered, and the operations were resumed.

**Table 5:** Triggering event Data are represented as n (%).

Triggering event	N (%)
Rod application and curve correction manoeuvre	5 (62.5)
Screw insertion	2 (25.0)
Corpectomy procedure	1 (12.5)

**Table 6:** Alert intervention Data are represented as n (%).

Alert intervention	N (%)
Removal of instrumentation	3 (37.5)
Release of curve correction	2 (25.0)
Raised blood pressure	2 (25.0)
Operation abandoned	1 (12.5)

All patients in the alert group did not experience new postoperative neurological deficits immediately after surgery or after six months. In the non-alert group, one patient developed a right-sided foot drop and reduced sensation from L5-S1 after surgery. SSEPs and MEPs were monitored throughout the surgery, and no alerts were experienced post-correction.

## Discussion

In this study, eight (3.7%) of 216 cases experienced IONM alerts during spinal deformity surgery, a rate that is consistent with the literature [11]. A recent study by Lee et al. reported that 2.1% of paediatric patients diagnosed with AIS demonstrated IONM signal changes [12]. Samdani et al. identified 5.3% of patients with AIS who experienced IONM alerts [3]. Kamerlink et al. reported that 4.6% of paediatric patients had changes in spinal cord monitoring leading to an alert [13]. 

Predictors of IONM alerts were assessed to aid surgeons in anticipating and managing intraoperative alert situations. The current study suggests that there is an association between IONM alerts and greater surgical extent. Kamerlink et al. identified an increased risk of IONM alerts in patients with higher BMI, EBL, operative time and postoperative thoracolumbar or lumbar coronal Cobb angle [13]. Our findings were broadly consistent with these observations, with patients experiencing alerts demonstrating longer operative times and greater preoperative and postoperative major Cobb angles. Although EBL was higher in the alert group, this difference did not reach statistical significance (p = 0.062). Nevertheless, increased blood loss and prolonged operative time have been proposed as potential contributors to spinal cord ischaemia and neurological injury. Yang et al. reported recovery of IONM signals in 20% of patients following an increase in MAP alone, rising to 60% when MAP augmentation was combined with other interventions [14]. Similarly, Kobayashi et al. suggested that excessive blood loss may contribute to MEP changes through spinal cord ischaemia, although systemic blood pressure may not always accurately reflect spinal cord perfusion [15]. Operative duration was significantly longer in the alert group, but this finding should be interpreted cautiously, as operative time was frequently extended by the additional time required to identify and respond to IONM alerts.

In the literature, Samdani et al. were the first study to analyse the radiographic and clinical outcomes of patients with IONM alerts [3]. They reported that the group of patients who experienced IONM alerts had a larger preoperative major Cobb angle. Among the patients who had a completed operation, the postoperative major Cobb angle and percentage of correction were similar between the alert and non-alert groups. Our study found a correlation between IONM alerts and larger preoperative and postoperative major Cobb angles. There was no significant difference in the mean corrective rate. A potential reason could be due to incomplete deformity corrections and the staging of operations following an alert, leading to an overall reduced degree of correction.

Historically, patients with greater preoperative deformities are at a higher risk of experiencing IONM alerts due to greater changes in spinal cord trajectory during deformity correction [16,17]. Similarly, procedures that involve changing the spinal cord trajectory increase the risk of cord injury. Kobayashi et al. reported that 64% of patients had exhibited an IONM alert during rotation manoeuvre, followed by 14% during incision, 7% after screw fixation, 7% during placement of the second rod and 7% after intervertebral compression [15]. Our study reported that five patients with IONM alerts were caused by rod application and curve correction manoeuvre, two caused by insertion of screws, and one caused by corpectomy procedure. Although this study identified more IONM alerts during and after insertion of instrumentation and curve correction manoeuvres, it is important to note that spinal cord monitoring should be performed with the same level of attentiveness throughout the correction, more so at the end of and after the correction. Following the study by Kamerlink et al. [13], attention is now also paid to spinal cord monitoring at the beginning of the surgery, as several reports have shown IONM alerts occurring before correction or without correction [18,19]. Where a true alert has been identified, it is essential that the triggering event is reversed. If signals fail to recover despite appropriate intervention, staging or aborting the procedure may be considered to minimise the risk of neurological injury. However, these management recommendations are based on established clinical practice rather than being directly demonstrated by the results of this study.

Out of the eight cases, one operation was aborted and staged. During an elective correction of idiopathic scoliosis, there was a loss of MEP signals in the left tibialis anterior. The patient's blood pressure was increased, but there was no change in signals. The surgeons loosened the end caps of the rods, which caused improvement in signals. Once they had returned to previous amplitudes, the surgeons proceeded to have a second attempt at correction (approximately 25 minutes following return of signals). Once again, there was a clear loss of left lower limb MEPs and a reduction in right lower limb MEPs. The surgeons loosened the caps and removed rods to facilitate spinal cord perfusion. The operation was abandoned due to signal loss on two occasions and was repeated two weeks later. The operation was successful after the second attempt, and the patient did not develop new neurological deficits immediately post-operation or after six months. Out of the remaining 208 non-alert cases, one patient developed postoperative neurological deficits following posterior scoliosis repair. This included a right-sided foot drop and reduced sensation in L5-S1. SSEPs and MEPs were monitored throughout the operation. Baseline lower limb SSEP and MEP responses from the right leg were poorly formed, whilst those from the left leg were clearly defined. The surgeons were informed and proceeded with the surgery. There were no significant changes from baseline throughout the operation and 20 minutes post-correction, which indicated that the dorsal column and corticospinal tract pathways were functionally intact. Follow-up nine weeks post-operation showed no sensory impairment or motor weakness. X-ray showed normal placement of metalwork with no complications, and MRI did not demonstrate nerve root compression.

This study has several limitations. First, its retrospective, single-centre design limits the generalisability of the findings and introduces the potential for selection and information bias. Second, the number of true positive IONM alerts was small, limiting statistical power and precluding reliable multivariable analysis. Therefore, the observed associations should not be interpreted as independent predictors. Third, missing data were present for some variables, particularly operative time, as these data were not routinely recorded in earlier cases. Analyses were performed using available data for each variable, resulting in the number of observations varying between analyses. Finally, multiple variables were assessed, increasing the potential for type I error, so results should be interpreted cautiously. Future multicentre prospective studies with larger cohorts and a greater number of IONM alerts are warranted to externally validate these findings and identify additional predictive factors for IONM alerts.

## Conclusions

In conclusion, this retrospective study identifies clinical factors associated with IONM alerts in paediatric spinal deformity patients, consistent with findings previously described in the literature. Patients experiencing IONM alerts had significantly longer operative times and greater preoperative and postoperative major Cobb angles. However, operative duration should be interpreted cautiously, as prolonged operative time may reflect additional time required for investigation and management of IONM alerts rather than representing an association for alert occurrence. IONM waveform changes most commonly occurred during or following instrumentation insertion and curve correction manoeuvres. In clinical practice, when an IONM alert occurs, the triggering event should be identified and reversed where possible to facilitate signal recovery. If signals cannot be recovered despite appropriate intervention, consideration may be given to staging or aborting the procedure to minimise the risk of neurological injury.

## References

[1] Bridwell KH, Lenke LG, Baldus C, Blanke K (1998). Major intraoperative neurologic deficits in pediatric and adult spinal deformity patients. Incidence and etiology at one institution. Spine (Phila Pa 1976).

[2] Scoliosis Research Society (SRS), 2019 2019 (2023). Intraoperative Neurophysiological Monitoring of Spinal Cord Function During Spinal Deformity Surgery. https://www.srs.org/Education/Quality-and-Safety-Library#statements.

[3] Samdani AF, Bennett JT, Ames RJ (2016). Reversible intraoperative neurophysiologic monitoring alerts in patients undergoing arthrodesis for adolescent idiopathic scoliosis: What are the outcomes of surgery?. J Bone Joint Surg Am.

[4] Rajappa D, Khan MM, Masapu D (2021). Multimodal intraoperative neurophysiological monitoring in spine surgeries: The experience at a spine centre through years. Asian Spine J.

[5] Wi SM, Park SM, Chang SY, Lee J, Kim SM, Chang BS, Kim H (2021). Surgical causes of significant intraoperative neuromonitoring signal changes in three-column spinal surgery. Asian Spine J.

[6] Zuccaro M, Zuccaro J, Samdani AF, Pahys JM, Hwang SW (2017). Intraoperative neuromonitoring alerts in a pediatric deformity center. Neurosurg Focus.

[7] British Society for Clinical Neurophysiology (BSCN), 2016 2016 (2023). Updated ANS/BSCN Guidelines for Neurophysiological Recordings of the Spinal Cord During Corrective Spinal Deformity. https://www.bscn.org.uk/data/files/Guidelines/IOM_guide2.pdf.

[8] British Society for Clinical Neurophysiology (BSCN), 2018 2018 (2025). Updated ANS/BSCN Guidelines for Neurophysiological Recordings of the Spinal Cord During Corrective Spinal Deformity Surgery. https://www.bscn.org.uk/data/files/Guidelines/IOM_guide2.pdf.

[9] Taniguchi M, Cedzich C, Schramm J (1993). Modification of cortical stimulation for motor evoked potentials under general anesthesia: Technical description. Neurosurgery.

[10] Pelosi L, Lamb J, Grevitt M, Mehdian SM, Webb JK, Blumhardt LD (2002). Combined monitoring of motor and somatosensory evoked potentials in orthopaedic spinal surgery. Clin Neurophysiol.

[11] Vitale MG, Moore DW, Matsumoto H (2010). Risk factors for spinal cord injury during surgery for spinal deformity. J Bone Joint Surg Am.

[12] Lee CS, Hwang CJ, Lee DH, Cho JH, Park S (2024). Risk factors and exit strategy of intraoperative neurophysiological monitoring alert during deformity correction for adolescent idiopathic scoliosis. Global Spine J.

[13] Kamerlink JR, Errico T, Xavier S (2010). Major intraoperative neurologic monitoring deficits in consecutive pediatric and adult spinal deformity patients at one institution. Spine (Phila Pa 1976).

[14] Yang J, Skaggs DL, Chan P (2018). Raising mean arterial pressure alone restores 20% of intraoperative neuromonitoring losses. Spine (Phila Pa 1976).

[15] Kobayashi K, Imagama S, Ito Z (2017). Transcranial motor evoked potential waveform changes in corrective fusion for adolescent idiopathic scoliosis. J Neurosurg Pediatr.

[16] Feng B, Qiu G, Shen J (2012). Impact of multimodal intraoperative monitoring during surgery for spine deformity and potential risk factors for neurological monitoring changes. J Spinal Disord Tech.

[17] Lewis SJ, Gray R, Holmes LM, Strantzas S, Jhaveri S, Zaarour C, Magana S (2011). Neurophysiological changes in deformity correction of adolescent idiopathic scoliosis with intraoperative skull-femoral traction. Spine (Phila Pa 1976).

[18] Leung YL, Grevitt M, Henderson L, Smith J (2005). Cord monitoring changes and segmental vessel ligation in the "at risk" cord during anterior spinal deformity surgery. Spine (Phila Pa 1976).

[19] Hsu B, Cree AK, Lagopoulos J, Cummine JL (2008). Transcranial motor-evoked potentials combined with response recording through compound muscle action potential as the sole modality of spinal cord monitoring in spinal deformity surgery. Spine (Phila Pa 1976).

